# Estimating the impact of prenatal health care services on adverse pregnancy outcomes in Tanzania: a propensity score matching approach

**DOI:** 10.3389/fgwh.2025.1589721

**Published:** 2025-07-22

**Authors:** Magashi Joseph Ntegwa, Riccardo Pelizzo

**Affiliations:** ^1^ Graduate School of Public Policy, Nazarbayev University, Astana, Kazakhstan; ^2^ Department of Geography and Economics, Dar es Salaam University College of Education, University of Dar es Salaam, Dar es Salaam, Tanzania

**Keywords:** antenatal care, stillbirths, miscarriage, adverse pregnancy, Tanzania, demographic and health survey, propensity score matching

## Abstract

**Introduction:**

The prevalence of adverse pregnancy outcomes remains one of the public issues that needs to be addressed in low- and middle-income countries (LMICs), including Tanzania. Despite evidence on the effectiveness of antenatal care (ANC) services in addressing adverse pregnancy outcomes, empirical studies are scarce. Therefore, this study aims to analyze the impact of ANC services on adverse pregnancy outcomes.

**Methods:**

This is a retrospective study that uses secondary data from the Tanzania Demographic and Health Survey for 2022. The impact of ANC services on adverse pregnancy outcomes was estimated using Propensity Score Matching (PSM), and the robustness of results was checked using doubly robust estimators

**Results:**

Adequate ANC services utilization reduces adverse pregnancy outcomes in Tanzania. Specifically, adequate ANC services utilization reduces adverse pregnancy outcomes: 5.6%–8.2% (depending on the PSM approach used). Similarly, an adequate ANC package reduces adverse pregnancy outcomes: 6.3%–9.3% (depending on the PSM approach used).

**Conclusions:**

The prevalence of adverse pregnancy outcomes needs to be addressed through adherence to ANC services utilization. Despite the utilization of ANC services being influenced by social, economic, and demographic factors, it is important to ensure essential packages of services are delivered to a pregnant woman for better pregnancy outcomes, as our results show that ANC visits alone have no significant impact.

## 1 Introduction

The purpose of the present study is to assess the impact of the utilization of antenatal care (ANC) services on adverse pregnancy outcomes in Tanzania. We use the 2022 Tanzania Demographic and Health Survey and Malaria Indicator Survey (2022 TDHS-MIS) data in the analysis.

There are at least three reasons why the study is worth conducting. The first reason is that adverse pregnancy outcomes remain a significant public health issue in the developing world and, especially, in Africa. The second reason is that while several studies have explored several determinants of adverse pregnancy outcomes, little attention has been paid to the extent to which ANC utilization can reduce the incidence of adverse pregnancy outcomes. In this respect one may note, as we will discuss in greater detail later on, that adverse pregnancy outcomes have been associated with the age of the mother, the religion of the parents, life in rural areas, educational attainment in the household and with the gender of the child, obstetric-related factors and, more importantly for the present study, with the distance from health care facilities. The third reason to focus on the Tanzania case is that about 10 per cent of pregnancies end up with adverse outcomes, which are not just problematic in themselves but may also have a wide range of negative consequences.

In this paper, we will proceed in two steps. We will first explore the determinants of ANC utilization, and we will then proceed to assess the impact of ANC utilization on adverse pregnancy outcomes. Our analyses reveal that the utilization of ANC is a function of the mother's age, education, literacy, place of residence and participation in decision making, household wealth, and both birth order and birth interval. Furthermore, consistent with what the literature had already established, our data analysis reveals that the utilization of ANC reduces adverse pregnancy outcomes in Tanzania.

The organization of the remainder of this paper is straightforward. In the first section, we will provide the background on adverse pregnancy outcomes and ANC, in the third section we will present our econometric framework for the analysis of the data on ANC utilization and adverse pregnancy outcomes, in the fourth we will discuss the data, the data sources and we will also present some descriptive statistics; in the fifth section we will present the results of our analysis and in the sixth we will formulate, as is customary, some conclusions and policy recommendations.

## 2 Background

Globally, adverse pregnancy outcomes remain a significant public health issue, especially in low-and middle-income countries (LMICs). The term “pregnancy outcomes” describes the result of a pregnancy, where adverse pregnancy outcomes include miscarriage, pre-term birth, or stillbirth, and live birth is considered a positive outcome (1). These outcomes constitute a significant risk factor for high rates of maternal morbidity, maternal near misses, and infant or child deaths. In 2020, approximately 13.4 million preterm births occurred globally, with rates ranging from 4% to 16% across countries (2). Also, preterm birth complications accounted for nearly 28.4 deaths per 100,000 population in 2019 (2, 3). In Tanzania, the preterm birth rate was estimated at 8.4 per 100 live births in 2020 (3).

Additionally, in 2021, approximately 1.9 million babies were stillborn at 28 weeks of pregnancy or later, with a global rate of 14 stillbirths per 1,000 births, 2.9 per 1,000 in Europe, Northern America, Australia and New Zealand region, 21 per 1,000 in sub-Saharan Africa, and 18 per 1,000 in Tanzania (4). Also, globally, it is estimated that 23 million miscarriages occur annually (5). Moreover, induced abortion is estimated to be more than 73 million annually in the world, with 45% of unsafe abortions occurring in LMIC settings (6). Unsafe abortion remains a primary cause of maternal deaths (6).

In Tanzania, in 2013, nearly 405,000 pregnant women had abortions, with the national abortion rate estimated at 36 per 1,000 women of reproductive age and a rate of 21 abortions per 1,000 live births (7). Moreover, in 2017, it was estimated that maternal abortion and miscarriage accounted for nearly 14% of maternal deaths in Tanzania (8). Furthermore, the 2022 Tanzania Demographic and Health Survey report indicates that a total of about 10% of pregnancies end up with adverse outcomes, with 0.2% (stillbirths), 8% (miscarriage), and 0.2% (induced abortion) (9). In 2020, Tanzania was among the ten countries that accounted for 60% of the burden in maternal deaths, neonatal deaths, and stillbirths (10). This leaves a lot to be desired if a country by 2030 is to reach 12 or less stillbirths per 1,000 total births and a neonatal rate as low as 12 deaths per 1,000 live births (11).

It is worth noting that adverse pregnancy outcomes such as pre-term births, miscarriage, abortion, and stillbirth lead not only to maternal and neonatal deaths but to long-term physical, neurodevelopmental, and trauma, high medical and healthcare costs, social impacts, and socioeconomic effects which have long-term personal and social impacts (12, 13). It is recommended that improved utilization of antenatal care during pregnancy reduces the likelihood of adverse pregnancy outcomes and pregnancy-related complications (14–16). Despite these recommendations, utilization of antenatal care faces different barriers in different contexts, resulting from individual, socioeconomic, and cultural factors that result in different pregnancy outcomes.

Scholars have examined different factors that increase the risk of adverse pregnancy outcomes. For instance, women who reside in rural areas have an increased risk of adverse pregnancy outcomes compared to their urban counterparts (17). On the other hand, the odds of adverse pregnancy outcomes decreased among women of middle and rich socioeconomic status compared to their poorer counterparts (18). Further, for women who attended secondary and above levels of education, their odds of adverse birth outcomes decreased (18). Additionally, it has been highlighted that female child is associated with a lower likelihood of adverse birth outcomes than males (18). Also, women who perceive the distance to the health facility as a big problem have higher odds of adverse birth outcomes (18). Older women were at high risk of adverse pregnancy outcomes (19). Similarly, systematic reviews showed a significant association between increased maternal age and stillbirth (20). Moreover, a study in Tanzania revealed that advanced maternal age is associated with higher odds of low birth weight, stillbirth, and neonatal intensive care unit transfer (21). Also, a non-religious or Muslim woman had a lower likelihood of adverse pregnancy outcomes (19), and being married or in a relationship increases the likelihood of adverse pregnancy outcomes (19).

Scholars have also identified obstetric-related factors, such as lack of ANC attendance, that are associated with increased risks of adverse birth outcomes (14, 17, 18, 22). Moreover, pregnancy-induced hypertension during pregnancy increases the risk of adverse pregnancy outcomes (17, 22). Also, anaemic mothers are at an increased risk of adverse pregnancy outcomes (14, 17, 22). Moreover, premature rupture of the membrane increases the risks of adverse pregnancy outcomes (22). Additionally, mothers with a history of adverse birth outcomes are at increased risk of stillbirth and adverse outcomes in subsequent pregnancies (23). Also, lack of dietary supplementation is associated with the development of adverse birth outcomes (22). Further, it was articulated that not receiving dietary counselling during ANC visits is associated with adverse birth outcomes (14). Moreover, physical abuse/abuse is associated with the development of adverse birth outcomes (18, 22). Also, no participation in healthcare decision-making is associated with increased adverse birth outcomes (18). Short birth intervals are associated with adverse birth outcomes (14, 24). Women with multiple pregnancies were more likely to have adverse birth outcomes (23, 24), and twin birth was associated with adverse birth outcomes (18). Further, not using family planning methods is associated with having stillbirths (14). Moreover, women who delivered through cesarean section were more likely to have stillbirths than women who had spontaneous vaginal delivery (23).

Efforts are in place to reduce adverse birth outcomes and their consequences globally and nationally. Intervention through maternal healthcare services, especially antenatal care services utilization, is emphasized. It is argued that early antenatal care visits, meeting the required number of ANC visits, and being attended by skilled personnel during antenatal visits are likely to reduce complications that would result in adverse pregnancy outcomes, as there will be close monitoring and the pregnant woman as adverse pregnancy outcomes are preventable. Thus, ANC utilization is critical as it reduces complications from pregnancy and childbirth, as well as reducing stillbirth and perinatal deaths (25). The World Health Organization (WHO) recommends a minimum of eight antenatal visits, health facility delivery, and skilled attendance for effective neonatal care (26). Also, it has been argued that emergency obstetric care and skilled birth attendants are the primary pathways to reducing maternal near-misses and deaths (27).

Studies on the impact of antenatal care visits on adverse birth outcomes are scarce. For instance, a study in Northwest Ethiopia showed a 0.10 and 0.11 decrease in adverse pregnancy outcomes as a result of the completion of a continuum of visit-based ANC and continuum of care via space dimension, respectively (28). Also, a systematic review and meta-analysis on the impact of antenatal care on maternal near-miss events in Ethiopia revealed that at least one ANC visit could avert three-fourths of maternal near-miss (29). Another study in Ethiopia showed that optimal antenatal care reduces perinatal deaths and improves maternal health (30). Moreover, women who attend at least one ANC visit are more likely to have live births compared to their counterparts (31). Other studies showed that completing ANC visits reduces preterm birth and low birth weight (14, 24, 32, 33). Additionally, receiving recommended ANC visits has a significant impact on preventing adverse pregnancy outcomes (14, 32, 33).

Adverse pregnancy outcomes can be managed easily if the utilization of antenatal care is adhered to as per WHO recommendations. However, according to the recent study by Zelka et al., the impact evaluation of the impact of ANC utilization on adverse pregnancy outcomes is extremely scarce. Studies on the utilization of ANC impact on adverse pregnancy outcomes are limited, with weak study design and analysis. Therefore, this study aims to contribute to the growing literature using an original country-wide survey data set to quantify the effect of ANC utilization on women's pregnancy outcomes. The effect of ANC utilization at the country level gives a broader perspective of analysis, which is necessary for policy design.

## 3 Econometric framework

### 3.1 Estimation strategy

Generally, a pregnant woman decides to utilize antenatal care services for the services that are provided, such as maternal and fetal health monitoring, immunization and preventive care, birth preparedness, and guidance on a healthy pregnancy. Thus, an assumed rational pregnant woman would choose to utilize antenatal care services if the expected utility from the utilization *M*_1_ is greater than that of not utilizing *M*_0_. The utility gain from the utilization of antenatal care services $M^{*}=M_{1}-M_{0}$ can be expressed as a function of an observable vector of covariates (*Z*) in a latent model as follows:

(1) $$M_{i}^{*}=\alpha \;Z_{i}+\mu_{i},\;\;\;\;\;\;\;\;M_{i}=1\;\mathrm{if}\;\;\;M_{i}^{*}>0,$$

where *M_i_* is a binary variable that equals 1 if a pregnant woman *i* utilized antenatal care services and zero otherwise; *α* is a vector of parameters to be estimated, and *Z_i_* is a vector of pregnant woman, household, and community level characteristics; and *μ_i_* is a random error term assumed to be normally distributed. Utilization of antenatal care services is expected to affect our outcome variable (i.e., adverse pregnancy outcomes). Assuming that our outcome variable is a linear function of a vector of exogenous variables *X_i_* and endogenous antenatal care services utilization *M_i_* such that:

(2) $$Y_{i}=\beta X_{i}+\delta M_{i}+\varepsilon_{i}$$

where *Y_i_* represents the outcome variable (adverse pregnancy outcomes); *M_i_* binary variable equals 1 if the pregnant woman utilized antenatal care services and zero otherwise; *β* and *δ* are the vector of parameters to be estimated, and *ε_i_* is the error term. However, pregnant women may self-select into the utilization of antenatal care, rather than being randomly selected. Thus, estimating Equation 2 using ordinary least squares (OLS) might produce biased estimates. We estimated the impact of antenatal care services utilization on adverse pregnancy outcomes using propensity score matching (PSM) because it controls for selection bias by controlling for observable confounding factors. However, an important shortcoming of using PSM is the inability to control hidden biases of the sampled unit. Therefore, we checked the robustness of our results using doubly robust estimation techniques and the sensitivity analysis of our estimates.

#### 3.1.1 Propensity score matching (PSM)

Utilization of antenatal care (our intervention) in Tanzania does not have any set of criteria and thus lacks randomization at the onset. Unlike in randomized control trials (RCTs), where participants are assigned randomly to either treatment or control group, in observational studies, randomization cannot be used to assign participants to either group. This results in an imbalance of the observed variables, which introduces bias and influences the causal effect of the exposure (34). A propensity score matching proposed by Rosenbaum and Rubin in 1983 reduces the bias in the estimation of treatment effects with observational data (35). Additionally, Rubin suggested that observational studies have to be designed to approximate random trials to obtain objective causal inference (36).

The study estimated the average treatment effects on treated using propensity score matching (PSM). PSM is a quasi-experimental technique that can be used to establish causal relationships in observational studies. According to Rosenbaum and Rubin, the PSM uses the observable characteristics of the sample to create a control group that is comparable to the treatment group, conditional on observable characteristics different from the intervention status, and in this study is the utilization of antenatal care services (35). In PSM, two assumptions need to be considered. The first is conditional independence (i.e., unconfoundedness), which requires that observable characteristics must be independent of potential outcomes, which implies that the decision to utilize antenatal care services is based on the observable characteristics of women, households, and community. The second assumption is that the common support condition needs to be satisfied, in which the distribution of observable characteristics between women who use antenatal care as recommended and women who don't use it needs to overlap.

Empirical estimation using PSM involves a series of steps. In the first step, we regressed the utilization of ANC on observable variables *Z* (as in Equation 1) to obtain the propensity scores using a logit estimation. Thus, the estimated propensity score $\left(PS_{i}=Prob(M_{i}=1|Z_{i})\right)$ represents the probability of pregnant women to utilization of ANC, and the marginal effects express the impact of variables in Z on this probability. We included several factors in Z to minimize omitted variables bias. Secondly, the obtained propensity scores (*PS*) are used to match women who used ANC adequately to women with inadequate use of ANC. Numerous algorithms can be applied to match ANC users and ANC-inadequate users. However, it has been noted that PSM methods are sensitive to a particular specification and matching methods (37, 38). Therefore, four different matching techniques: nearest neighbor matching (NNM), kernel matching, radius matching, and stratification matching methods were used in the estimations. In the third step, we assess the overall covariates' balancing property and the overlap over the common support. The final step involves calculating the Average Treatment Effect in treated (ATET), which is the mean difference between the two matched groups (38, 39).

Therefore, the estimated ATET is as follows:

(3) $$ATET(Z)=E[Y_{1}|M=1,\;\mathrm{Prob}\;(Z)]-E[Y_{0}|M=1,\;\mathrm{Prob}\;(Z)],$$

Where *Y*_1_ represents the outcomes indicator of the ANC utilization, and *Y*_0_ is the outcome indicator for the inadequate use of ANC; *M* is a binary variable that equals 1 if a pregnant woman utilized antenatal care services and zero otherwise

However, since matching main assumption is selection on observables, our estimates can be sensitive to unobserved bias, which could result in positive and negative unobserved selection. Therefore, we checked for the sensitivity of our estimated treatment effect to hidden bias using the Rosenbaum bound approach (40). The Rosenbaum approach considers the sensitivity parameter Г that measures the degree of departure from random assignment of treatment. This implies that two women with similar observed characteristics may differ in the odds of utilizing ANC by at most a factor of Г. The Mantel-Haenzel (MH) test statistic was used to check if our PSM estimates are sensitive to the hidden bias because our outcome variables are binary (41). We use gamma coefficients as the factor by which hidden bias affects the assignment of the intervention to the treated and control groups. We use the gamma value that ranges from 1 to 2 with an increment of 0.05 using the ***mhbounds*** STATA command (41). All analyses were conducted using STATA 18 (42).

#### 3.1.2 Doubly robust estimator

Although we conducted our analysis using different PSM approaches, PSM relies on having a correctly specified model. We then estimated the average treatment effect on the treated using doubly robust estimators, including inverse-probability weighted regression adjustment (IPWRA) and augmented inverse probability weighting (AIPW). Despite estimating ATET using PSM models, biased results can be yielded because of misspecification of the model (43, 44). Scholars have proposed the adoption of an inverse probability weighted (IPW) technique to address this issue. Because of its double-robust property, IPW allows both the outcome and the treatment model to correct for misspecification and yields consistent findings (43–45). Moreover, doubly robust estimators reduce selection bias from non-random treatment assignments by modelling the treatment and outcome simultaneously (46). Therefore, we estimated ATET using both the augmented inverse probability weighting (AIPW) and inverse probability weighted regression adjustment (IPWRA) models. The treatment effect of these models is estimated using weighted regression coefficients, where the weights are the predicted inverse probabilities of treatment (45).

According to Pinzón, estimating the treatment effect of utilization of ANC on adverse pregnancy outcomes using AIPW and IPWRA follows three steps, as explained here for each (47). In the AIPW, the first step is estimating the treatment model and computing the inverse probability weight. The second step involves estimating a separate regression model of the outcome for each treatment level. The third step involves computing the weighted means of the treatment-specific predicted outcomes, where the weights are the inverse probability computed in the first step. Also, the three steps for estimating treatment effects of utilization of ANC on adverse pregnancy outcomes using IPWRA include the first step is to estimate the probability of utilizing ANC (i.e., a treatment model) and compute inverse-probability weights using the predicted probabilities. In the second step, use the estimated inverse probability weights to fit weighted regression models to obtain expected outcomes of the probabilities of utilizing ANC or not utilizing ANC. Lastly, to compute the means of utilizing and not utilizing ANC, and the difference provides the estimates of the treatment effect of the user and non-user of ANC. The double-robust property of the IPWRA and AIPW approaches is their key feature; it enables the treatment effect to be reliably estimated as long as either the outcome model or the treatment model is specified correctly (45, 46). Equation 3, which is used in the estimation using the PSM methods, also follows when estimating the average treatment effect of treated (ATET) of ANC utilization on adverse pregnancy outcomes, using the doubly robust estimators (i.e., AIPW and IPWRA).

### 3.2 Data source and descriptive statistics

#### 3.2.1 Data sources

The study used cross-sectional data from the 2022 Tanzania Demographic and Health Survey (TDHS), a nationally representative survey conducted by the National Bureau of Statistics (NBS) and the Office of the Chief Government Statistician, Zanzibar (OCGS). Technical assistance was provided by ICF through the DHS Program, funded by the United States Agency for International Development (USAID). The 2022 TDHS-MIS sample design was conducted in two stages to provide estimates for the entire country. The first stage involves selecting sampling points (clusters) composed of the enumeration areas (EAs) delineated during the 2012 Tanzania Population and Housing Census. EAs were chosen with probability proportional to their size within each sampling stratum, resulting in a selection of 629 clusters. In the second stage, 26 households were systematically selected from each cluster, for an anticipated sample size of 16,354 households. The 2022 TDHS-MIS used five questionnaires: the household questionnaire, the women's questionnaire, the biomarker questionnaire, and the micronutrient questionnaire. The woman's questionnaire, which is the focus of this study, collects demographic, health, and nutrition information on women and children. Further details on sampling and questionnaires are available in the TDHS-MIS main report (9). According to the 2022 TDHS-MIS, 15,699 women were eligible for interviews; however, 15,254 women were successfully interviewed (97% response rate). However, only 7,281 respondents had given birth in the last five years before the survey. Additionally, only 6,220 respondents had responses to the pregnancy outcomes questions. After data cleaning and removing observations with no information on the outcome and covariates, a final sample of 4,665 women was used for the analysis.

#### 3.2.2 Descriptive statistics

Table 1 shows the descriptive statistics of the variables used in the analysis for the sampled women. Additionally, it reports the differences in statistics between variables based on whether the pregnant woman had adequate ANC or not. Our main independent variable, “antenatal care services utilization”, is whether the pregnant woman received the recommended antenatal care (i.e., ANC package) and whether she had the recommended antenatal visits (i.e., ANC visits) and then adequate ANC if utilized all the ANC package and had all the recommended ANC visits. On one hand, the pregnant woman meets the recommended ANC package if during her pregnancy she had a tetanus toxoid injection, iron tablets/syrup, a blood sample taken, a urine sample taken, and blood pressure taken. This was coded as equal to 1 if the woman had all during her pregnancy and zero if she missed any. On the other hand, ANC visits were coded as equal to 1 if the pregnant woman had her first ANC within the first trimester, had four or more ANC visits, and had at least one ANC provided by a skilled provider, and zero if she missed any. Finally, we coded the variable adequate ANC equal to 1 if the pregnant woman had 1 in the ANC visits and ANC package, and zero otherwise. As shown in Table 1, only 12% of pregnant women had adequate ANC care. The construction of our variable of interest is as per the WHO recommendation for the care of pregnant women (25).

**Table 1:** Summary statistics of variables.

Variables	Pooled	Adequate ANC	Inadequate ANC	*p*-value
Adequate ANC (1 = yes, 0 = no)	0.124 (0.330)			
Adverse Pregnancy Outcomes (1 = yes, 0 = no)	0.059 (0.236)	0.066 (0.249)	0.010 (0.101)	<0.01
Woman age	30.565 (6.475)	30.630 (6.558)	30.118 (5.879)	0.067
Woman age square	976.166 (413.139)	981.170 (418.538)	941.578 (373.551)	0.067
Women's education (1 = no education)	0.217 (0.412)	0.234 (0.423)	0.106 (0.308)	<0.01
Women's education (2 = primary education)	0.531 (0.499)	0.531 (0.499)	0.531 (0.499)	0.999
Women's education (3 = sec or higher)	0.252 (0.434)	0.235 (0.424)	0.363 (0.481)	<0.01
Partner education (1 = no education)	0.146 (0.353)	0.156 (0.363)	0.076 (0.265)	<0.01
Partner education (2 = primary education)	0.588 (0.492)	0.598 (0.490)	0.521 (0.500)	<0.01
Partner education (3 = sec or higher)	0.266 (0.442)	0.246 (0.431)	0.403 (0.491)	<0.01
Wealth status (1 = poorest)	0.222 (0.415)	0.237 (0.425)	0.114 (0.318)	<0.01
Wealth status (2 = poorer)	0.202 (0.402)	0.211 (0.408)	0.145 (0.353)	<0.01
Wealth status (3 = Middle)	0.199 (0.400)	0.205 (0.404)	0.163 (0.369)	0.017
Wealth status (4 = Richer)	0.195 (0.396)	0.190 (0.392)	0.234 (0.423)	0.013
Wealth status (5 = Richest)	0.182 (0.386)	0.158 (0.364)	0.344 (0.476)	<0.01
Birth order (≥4 = yes)	0.554 (0.497)	0.575 (0.494)	0.408 (0.492)	<0.01
Short birth interval (1 = yes)	0.223 (0.416)	0.229 (0.420)	0.182 (0.386)	0.01
Women's employment (1 = yes)	0.661 (0.473)	0.653 (0.476)	0.715 (0.452)	<0.01
Household head (1 = male)	0.869 (0.337)	0.871 (0.335)	0.856 (0.351)	0.319
Wife beating justified (1 = yes)	0.479 (0.500)	0.490 (0.500)	0.408 (0.492)	<0.01
Decision making (1 = yes)	0.523 (0.500)	0.512 (0.500)	0.600 (0.490)	<0.01
Media exposure (1 = yes)	0.640 (0.480)	0.620 (0.485)	0.777 (0.417)	<0.01
Residence (1 = rural)	0.752 (0.432)	0.777 (0.416)	0.581 (0.494)	<0.01
Community poverty (1 = high)	0.500 (0.500)	0.526 (0.499)	0.324 (0.468)	<0.01
Community media access (1 = high)	0.466 (0.499)	0.442 (0.497)	0.625 (0.485)	<0.01
Community literacy (1 = high)	0.380 (0.485)	0.354 (0.478)	0.554 (0.498)	<0.01
Observations	4,665	4,087 (0.88)	578 (0.12)	

Our outcome variable is adverse pregnancy outcomes. It is measured by the self-reports of women of reproductive age on whether they experienced stillbirth, miscarriage, or abortion. Therefore, we coded as “1” if a woman had an adverse pregnancy outcome and “0” if she reported a live birth. This is as per the definition provided by Magadi and colleagues (1). In the sample size used in this study, nearly 6% of the pregnant women had adverse pregnancy outcomes (see Table 1).

Based on the existing literature on adverse pregnancy outcomes, our model included several control variables such as respondents and household characteristics (age, education, household head sex, household wealth status, birth order, birth interval, employment, wife beating, media exposure and decision making) and community characteristics (place of residence, proportion of women literacy in the community, women media exposure in the community, and community poverty). The average age of the woman is 30 years, with the majority (over 50%) having the primary level of education. Also, the majority of husbands had the primary level of education (i.e., 58%). Moreover, there was high justification for wife-beating (48%), and participation in decision-making was 52%. Further, one in five had a short birth interval (i.e., 22%), with the majority having more than four birth orders (55%). Also, the majority of households are male-headed, and the majority reside in rural areas (75%). Additionally, while the community media access is at 47%, the majority of the community had the highest poor households at 50% with women having the lowest literacy rate at only 38%.

Table 1 also presents the results for the differences between women who had adequate ANC use and those without. There are evident significant differences between the two groups. For instance, compared to the control groups, women in the treatment group had significantly lower women and partners with no education and higher rates of women and partners with secondary/above education. Also, there were significantly fewer women with short birth intervals and four or more birth orders. Moreover, there were significantly fewer women who justified women beating and significantly higher numbers who participated in decision-making. The presented results, together with descriptive statistics for different treatments (ANC package and ANC Visits), are shown in Supplementary Tables A.1, A.2 in the Appendices. This indicates that systematic differences between the treatment and control groups and between women in different intervention categories exist. Therefore, estimation using the PSM and doubly robust estimators in estimating average treatment effects is considered the best.

Moreover, Table 1 presents the mean differences of the outcome variable (i.e., adverse pregnancy outcome) between those who had adequate ANC and those who missed some. It shows that, on average, adequate ANC utilization had significantly lower adverse pregnancy outcomes (1%) than those who did not or had few (6.6%), with a pooled average of 5.9%. The significant difference provides evidence that adequate ANC had a lower adverse pregnancy outcome than their counterparts. However, the mean comparison does not provide any conclusions and cannot be used to explain the relation between ANC utilization and adverse pregnancy outcomes, as it does not consider potential selection bias.

## 4 Results and discussion

This section reports the factors that determine the utilization of antenatal care services using the logit regression model. Then, the results of the impact of antenatal care utilization on adverse pregnancy outcomes using different propensity score matching methods are presented. Also, the robustness of the obtained results is estimated using the doubly robust estimators. Finally, sensitivity analyses are presented and discussed.

### 4.1 Determinants of ANC utilization: logistic estimates

Factors that determine women's decision to utilize ANC are presented in Table 2 with their marginal effects. The likelihood ratio test indicates that the model estimates are significant at a 1% level [χ^2^ = 237.98 (21), *p* < 0.01]. This implies that our model is statistically significant, and our covariates jointly and significantly explain pregnant women's decision to utilize ANC services. Results indicate that women's age, education, household wealth status, birth order, short birth interval, women's employment, women's participation in decision-making, and place of residence are significantly associated with the utilization of adequate ANC (column 1).

**Table 2:** Logit estimation of determinants of ANC utilization.

Variables	Adequate ANC		ANC Package		ANC visits	
	Coefficients	Marginal effects	Coefficients	Marginal effects	Coefficients	Marginal effects
Woman age	0.148 (0.073)***	0.015 (0.008)***	0.040 (0.049)	0.008 (0.010)	0.178 (0.055)***	0.035 (0.011)***
Woman age square	−0.002 (0.001)**	−0.000 (0.000)**	−0.001 (0.001)	0.000 (0.000)	−0.003 (0.001)***	−0.001 (0.000)***
Women primary education	0.389 (0.156)**	0.040 (0.016)**	0.195 (0.097)**	0.039 (0.019)**	0.310 (0.107)***	0.060 (0.021)***
Women with secondary/above education	0.153 (0.191)	0.016 (0.020)	0.075 (0.128)	0.015 (0.025)	0.274 (0.142)	0.053 (0.028)
Partner primary education	0.073 (0.180)	0.007 (0.019)	0.028 (0.112)	0.005 (0.022)	−0.075 (0.122)	−0.015 (0.024)
Partner secondary/above education	0.215 (0.205)	0.022 (0.021)	0.263 (0.135)*	0.052 (0.027)*	−0.027 (0.149)	−0.005 (0.029)
Poorer wealth status	0.226 (0.176)	0.023 (0.018)	0.035 (0.113)	0.007 (0.022)	0.294 (0.122)**	0.057 (0.024)**
Middle wealth status	0.164 (0.193)	0.017 (0.020)	−0.042 (0.125)	−0.008 (0.025)	0.256 (0.136)*	0.050 (0.026)*
Richer wealth status	0.326 (0.215)	0.034 (0.022)	0.179 (0.144)	0.035 (0.029)	0.248 (0.158)	0.048 (0.031)
Richest wealth status	0.615 (0.240)**	0.063 (0.025)**	0.221 (0.167)	0.044 (0.033)	0.484 (0.185)***	0.094 (0.036)***
≥4th birth order	−0.546 (0.118)***	−0.056 (0.012)***	−0.361 (0.087)***	−0.071 (0.017)***	−0.459 (0.095)***	−0.089 (0.018)***
Short birth interval	−0.237 (0.121)**	−0.024 (0.012)**	−0.213 (0.083)**	−0.042 (0.016)**	−0.087 (0.095)	−0.017 (0.019)
Women employed	0.306 (0.103)***	0.031 (0.011)***	0.312 (0.072)***	0.062 (0.014)***	0.349 (0.081)***	0.068 (0.016)***
Male household head	−0.064 (0.133)	−0.007 (0.014)	−0.084 (0.097)	−0.017 (0.019)	−0.021 (0.107)	−0.004 (0.021)
Wife beating justified	−0.117 (0.096)	−0.012 (0.010)	−0.016 (0.069)	−0.003 (0.014)	−0.055 (0.075)	−0.011 (0.015)
Participation in decision-making	0.183 (0.095)*	0.019 (0.010)*	0.019 (0.069)	0.004 (0.014)	0.085 (0.075)	0.017 (0.015)
Media exposure	0.168 (0.126)	0.017 (0.013)	0.091 (0.084)	0.018 (0.017)	0.274 (0.092)***	0.053 (0.018)***
Rural residence	−0.287 (0.127)**	−0.030 (0.013)**	−0.528 (0.095)***	−0.104 (0.019)***	0.054 (0.108)	0.011 (0.021)
Poor households in the community	−0.020 (0.158)	−0.002 (0.016)	−0.132 (0.110)	−0.026 (0.022)	−0.184 (0.116)	−0.036 (0.023)
Community media access	0.078 (0.121)	0.008 (0.012)	0.154 (0.086)*	0.030 (0.017)*	−0.152 (0.093)	−0.030 (0.018)
Community literacy	0.296 (0.128)**	0.030 (0.013)**	0.380 (0.095)***	0.075 (0.019)***	0.049 (0.103)	0.010 (0.020)
Constant	−4.807 (1.154)***		−1.270 (0.783)		−4.1716 (0.8686)	
Pseudo R^2^	0.068		0.0758		0.0378	
Model χ^2^	237.98		414.23		177.2	
*p*-value	0.000		0.0000		0.0000	
Log likelihood	237.98		−2,709.0126		−2,255.451	
Observations	4,658		4,665		3,922	

Robust standard errors in parentheses.

* *p* < 0.1.

** *p* < 0.05.

*** *p* < 0.01.

The age of the pregnant woman exerts a positive and significant impact on adequate ANC utilization, indicating that older women are 1.5 percentage points more likely to utilize adequate ANC than their counterparts' younger counterparts. Women's primary education variable was positive and significant, indicating that women with primary education are 4 percentage points more likely to utilize adequate ANC than women with no education. These results are supported by previous studies (48, 49). Also, household wealth status has a positive and significant impact on antenatal care services utilization. For instance, compared to the women from the poorest households, women from the richest households are 6.3 percentage points more likely to utilize adequate ANC. These findings corroborate those of (48, 50). According to Zhang and Lu, in China, higher household income has a significant and positive association with the examination rate. Also, Tesfaye et al. in Ethiopia, women who belonged to higher wealth quintiles were more likely to attend at least one ANC visit.

Moreover, women with four or more birth orders are 5.6 percentage points less likely to utilize adequate ANC compared to those with less than four. Similarly, women with short birth intervals were 2.4 percentage points less likely to utilize adequate ANC compared to women with longer birth intervals. Additionally, women who were employed, from a community with high literacy, and who participated in decision-making were respectively 3.1, 3.0, and 1.9 percentage points more likely to utilize adequate ANC than their counterparts. Further, women from rural areas were also less likely to use adequate ANC compared to women from urban areas.

Moreover, Table 2 indicates different determinants of utilization of the ANC package (column 2). Women's education, husband's education, birth order, short birth intervals, employment, place of residence, and women's community literacy were found to significantly affect ANC package utilization. It is indicated that women with primary education were more likely to utilize the full ANC package compared to their counterparts, women with no education. Similarly, women with husbands with secondary/above levels of education are more likely to utilize the ANC package. Women with fourth or above birth order and short birth intervals are less likely to utilize the ANC package compared to their counterparts with less than four birth orders and longer birth intervals, respectively. Employed women were more likely to utilize the ANC package than unemployed women. Similarly, women from the community with women with high literacy were more likely to utilize the ANC package. However, women from rural areas were less likely to utilize the ANC package compared to women from urban areas.

Also, Table 2 shows the determinants of ANC visits (column 3). Similarly, the results for adequate ANC and ANC package, women's age are positive and significant. This implies that older women are more likely to meet the ANC visit components compared to the younger ones. Additionally, compared to women with no education, women with primary education levels are more likely to meet the ANC package. Moreover, compared to women from the poorest quintile, women from the poorer, middle, and richest quintiles are more likely to have ANC visits. Women who are employed and with media exposure are more likely to utilize the recommended ANC visits than their counterparts. However, women with four or more birth orders were less likely to meet the recommended ANC visits compared to women with less than four birth orders.

### 4.2 Impacts of ANC utilization on adverse pregnancy outcomes: PSM estimator

This section presents the treatment effects estimated from the PSM models. Based on the logit estimation, propensity scores were obtained for the matching. The validation of PSM models depends on the quality of the matching. Table 3 provides results for the overall balancing test of covariates. Results indicate that the standardized mean bias for all covariates used in the matching reduces from 22.9% to 2.6% (ANC package), 47.7% to 0.0% (ANC visits), and 67.8% to 0.0% (adequate ANC). Additionally, the likelihood ratio test shows that the null hypothesis of the joint significance of all covariates could be rejected before matching (*p* *>* χ^2^ = 0.000). Also, after the matching, the test of joint significance of all covariates could be rejected with *p* > χ^2^ = 0.662 (ANC package) and *p* *>* χ^2^ = 0.999 for ANC visits and adequate ANC. The low pseudo-*R^2^*, mean standardized bias, and the insignificant LR χ^2^ shown in Table 3 indicate the quality of our matching (37). Therefore, estimation of the average treatment effects can be done after a successful matching between the treatment and control groups. Also, Figures A.1–A.3 in the Appendix show the common support between the two groups. This implies that there is sufficient overlap in the distribution of the propensity scores across different ANC utilization aspects, indicating that the common support condition is satisfied. This indicates that there was a common support between the treated and control groups, and therefore, women who utilized the ANC and those who did not were similar based on the observables. Additionally, because of the common support option, one observation was dropped when we fitted the propensity-matched analysis on the impact of ANC package utilization on adverse pregnancy outcomes. Also, no observations were dropped for adequate ANC, while two were dropped for ANC visits (see Supplementary Table A.3) in the Appendix.

**Table 3:** Propensity score matching quality test.

ANC use	Before matching	After matching
ANC package		
Pseudo R^2^	0.076	0.004
LR χ^2^	444.72	7.79
*p* > χ^2^	0.000	0.662
Mean standardized bias	22.9	2.6
ANC visits		
Pseudo R^2^	0.037	0.000
LR χ^2^	175.79	0.00
*p* > χ^2^	0.000	0.999
Mean standardized bias	47.7	0.0
Adequate ANC		
Pseudo R^2^	0.066	0.000
LR χ^2^	231.3	0.00
*p* > χ^2^	0.000	0.999
Mean standardized bias	67.8	0.0

Table 4 shows the average treatment effect on the treatment group by the PSM models. Four matching methods were used: Nearest Neighbor Matching, Kernel Matching, Radius Matching, and Stratification. The average treatment effects on the treated for adverse pregnancy outcomes are all negative and statistically significant for adequate ANC and ANC packages, but not for the average treatment effect for ANC visits. The effect of the ANC package was evaluated at 9.3%, 9.0%, 6.3% and 9.1% in lowering adverse pregnancy outcomes. Overall, results are consistent in terms of signs and significance level and can be compared across the estimation techniques used. For instance, the ANC package is associated with a decrease in the likelihood of adverse pregnancy outcomes, which ranges between 6.3% by radius estimation techniques to 9.3% using the Nearest Neighbor Matching method. This implies that the ANC package reduces the likelihood of adverse pregnancy outcomes among pregnant women between 6% and 9%. On the other hand, ANC visits show an insignificant likelihood of reducing adverse outcomes. Moreover, adequate ANC reduces the likelihood of adverse pregnancy outcomes from 5.6% (radius estimation) to 8.2% (Nearest Neighbor). Therefore, the implication is that it is not about the number of visits that translates into a reduction of adverse pregnancy outcomes, but the package of services that the pregnant woman receives. A similar study Overall utilization of ANC services decreases the likelihood of adverse pregnancy outcomes among women. Therefore, from these results, it can be concluded that in the absence of observable selection bias, ANC package and adequate ANC utilization negatively and significantly affect adverse pregnancy outcomes. Our findings are corroborated by other studies that empirically reported a significant and negative relationship between antenatal care services utilization and adverse pregnancy outcomes in Ethiopia (28, 51), Ghana (52), and North Carolina (53). Thus, improved quality of ANC can improve pregnancy outcomes by providing preventive measures and encouraging health facilities to deliver, where pregnancy-related complications can be addressed (52). Additionally, the provision of primary health care services and social services reduces adverse pregnancy outcomes (54). Moreover, evidence in the UK demonstrates that completion of core intervention in the health system reduces preterm births and improves the survival of the neonates (55).

**Table 4:** ATET of antenatal care: PSM estimates.

Estimator	Observations	ATET
ANC package		
Nearest neighbor matching	2,585	−0.093 (0.012)***
Kernel matching	4,655	−0.090 (0.008)***
Radius matching	4,561	−0.063 (0.006)***
Stratification matching	4,655	−0.091 (0.008)***
ANC visits		
Nearest neighbor matching	1,952	−0.005 (0.006)
Kernel matching	3,922	−0.000 (0.004)
Radius matching	3,827	0.001 (0.004)
Stratification matching	4,665	−0.001 (0.004)
Adequate ANC		
Nearest neighbor matching	1,141	−0.082 (0.015)***
Kernel matching	4,647	−0.065 (0.007)***
Radius matching	4,466	−0.056 (0.006)***
Stratification matching	4,654	−0.070 (0.007)***

Standard errors in parentheses.

* *p* < 0.1.

** *p* < 0.05.

*** *p* < 0.01.

### 4.3 Robustness checks: doubly robust estimator

The robustness of our estimated ATET results was tested using two doubly robust estimators, IPWRA and AIPW (43, 46). The results are shown in Table 5. Results are consistent with the results obtained using PSM estimators, showing that our estimates are robust and not sensitive to the estimation methods used. Overall estimates from IPWRA and AIPW are consistent and the same across treatment groups. For instance, using both methods, adequate ANC reduces the likelihood of adverse pregnancy outcomes by about 5.7% for all the estimation methods. Also, the ANC package reduces the likelihood of adverse pregnancy outcomes by 7.4% across the estimation methods. Moreover, similar to the PSM estimation, ANC visits were found to reduce the likelihood of adverse pregnancy but insignificant. This implies that women's antenatal visits do not significantly reduce the likelihood of adverse pregnancy outcomes, but the ANC package does.

**Table 5:** ATET of antenatal care: doubly robust estimates.

Estimator	Observations	ATET
ANC package		
IPWRA	4,655	−0.07407 (0.0064)***
AIPW	4,655	−0.07410 (0.0064)***
ANC visits		
IPWRA	3,922	−0.00067 (0.0040)
AIPW	3,922	−0.00070 (0.0040)
Adequate ANC		
IPWRA	4,658	−0.0568 (0.0064)***
AIPW	4,658	−0.0570 (0.0064)***

Standard errors in parentheses.

* *p* < 0.1.

** *p* < 0.05.

*** *p* < 0.01.

### 4.4 Sensitivity analysis

The reliability of the estimates on propensity score matching depends on the selection of observables, and therefore, results can be biased if there is unobserved heterogeneity (i.e., hidden bias) between women who utilized ANC and those without ANC that affects the outcomes of interest. Therefore, we check the sensitivity of the ATET estimates to unobserved heterogeneity by computing the Rosenbaum sensitivity test (41). Table 6 shows that our results are robust to unobserved bias as both directions (positive and negative) remain significant across all tested levels of *Γ*. In Table 6 the analysis shows that when there is no hidden bias, that is, where Г = 1, the Q_MH_ statistic is 5.561 for adequate ANC and 9.47 for ANC package, and it constitutes strong evidence that adequate ANC and ANC package reduce adverse pregnancy outcomes. Moreover, the upper bound and lower bound on the significance level from Г = 1.05 to Г = 2 were significant and indicated that the study is not sensitive to hidden bias. The Mantel-Haenszel bounds show that the positive association becomes statistically significant at Г = 1.35 (p__MH+_ < 0.05) and strengthens to p__MH+_ = 0.002678 at Г = 2, indicating robustness to unmeasured confounding up to this level. Meanwhile, p__MH−_ remains non-significant across all Г, confirming no evidence of a negative association and highlighting the stability of the positive effect.

**Table 6:** Sensitivity analysis using Mantel-Haenszel bounds.

Gamma (Г)	Adequate ANC				ANC package				ANC visits			
	Q_mh+	Q_mh−	p_mh+	p_mh−	Q_mh+	Q_mh−	p_mh+	p_mh−	Q_mh+	Q_mh−	p_mh+	p_mh−
1	5.561	5.561	0.000	0.000	9.47	9.47	0.000	0.000	0.88	0.88	0.188	0.188
1.05	5.737	5.394	0.000	0.000	9.78	9.18	0.000	0.000	1.01	0.76	0.156	0.224
1.1	5.905	5.234	0.000	0.000	10.07	8.90	0.000	0.000	1.14	0.64	0.128	0.262
1.15	6.068	5.084	0.000	0.000	10.35	8.63	0.000	0.000	1.25	0.52	0.105	0.301
1.2	6.226	4.943	0.000	0.000	10.63	8.39	0.000	0.000	1.37	0.41	0.086	0.340
1.25	6.380	4.809	0.000	0.000	10.90	8.15	0.000	0.000	1.48	0.31	0.070	0.380
1.3	6.531	4.681	0.000	0.000	11.16	7.93	0.000	0.000	1.58	0.20	0.057	0.419
1.35	6.678	4.561	0.000	0.000	11.41	7.72	0.000	0.000	1.68	0.11	0.046	0.457
1.4	6.821	4.445	0.000	0.000	11.66	7.52	0.000	0.000	1.78	0.01	0.037	0.495
1.45	6.962	4.336	0.000	0.000	11.91	7.32	0.000	0.000	1.88	−0.08	0.030	0.531
1.5	7.099	4.231	0.000	0.000	12.15	7.14	0.000	0.000	1.97	−0.16	0.024	0.565
1.55	7.234	4.130	0.000	0.000	12.38	6.96	0.000	0.000	2.06	−0.14	0.020	0.555
1.6	7.366	4.034	0.000	0.000	12.61	6.79	0.000	0.000	2.15	−0.06	0.016	0.523
1.65	7.495	3.941	0.000	0.000	12.84	6.63	0.000	0.000	2.23	0.02	0.013	0.491
1.7	7.623	3.852	0.000	0.000	13.06	6.47	0.000	0.000	2.32	0.10	0.010	0.461
1.75	7.748	3.766	0.000	0.000	13.28	6.32	0.000	0.000	2.40	0.17	0.008	0.431
1.8	7.871	3.683	0.000	0.000	13.49	6.17	0.000	0.000	2.48	0.25	0.007	0.403
1.85	7.991	3.604	0.000	0.000	13.70	6.03	0.000	0.000	2.56	0.32	0.005	0.376
1.9	8.110	3.527	0.000	0.000	13.91	5.90	0.000	0.000	2.64	0.39	0.004	0.350
1.95	8.228	3.452	0.000	0.000	14.11	5.77	0.000	0.000	2.71	0.45	0.003	0.325
2	8.343	3.380	0.000	0.000	14.31	5.64	0.000	0.000	2.78	0.52	0.003	0.302

Gamma: odds of differential assignment due to unobserved factors; Q_mh+: Mantel-Haenszel statistic (assumption: overestimation of treatment effect); Q_mh-: Mantel-Haenszel statistic (assumption: underestimation of treatment effect); p_mh+: significance level (assumption: overestimation of treatment effect); p_mh-: significance level (assumption: underestimation of treatment effect).

## 5 Conclusions

The prevalence of nearly 10% adverse pregnancy outcomes in Tanzania threatens the efforts aimed at addressing pregnancy-related deaths, morbidity, and other complications. Also, while Tanzania has recently experienced a significant decrease in maternal mortality, stillbirths, miscarriages, and abortions are public health issues that need to be addressed. Evidence recommends that ANC utilization results in positive pregnancy outcomes. Our study results report that women's age, education, birth order, birth interval, employment, participation in decision making, household wealth status, place of residence, and women's literacy in the community influence pregnant women's decision to utilize antenatal care services. Our results also indicate that antenatal care services (ANC package and adequate ANC) reduce adverse pregnancy outcomes. Our results support the hypothesis that antenatal care services have the potential to reduce adverse pregnancy outcomes through the close monitoring of the pregnancy during pregnancy, delivery, and postpartum periods. These would ensure that the pregnant woman has a healthy pregnancy and reduce the likelihood of adverse pregnancy outcomes and other pregnancy-related complications.

Our findings have obvious policy implications. If, as we have shown, adverse pregnancy outcomes are affected by the utilization of ANC, then governments, starting with the Tanzanian government, should make a greater effort to promote the utilization of ANC services and ensure services are adequately provided as per the recommendations by WHO (adequate ANC package). This requires government investment (through increased budget) to implement the maternal and child health policy, focusing on workforce expansion, supply chains, and facility readiness to ensure all pregnant women receive complete, high-quality ANC services regardless of geographic location or socioeconomic status. Moreover, since our analysis identified some factors that act as a barrier to ANC utilization, the government should address them to ensure that women have access to adequate services regardless of their socioeconomic characteristics (education, place of residence, and economic status). Taking these into consideration, policy makers would be in a position barriers to ANC utilization and finally address adverse pregnancy outcomes.

This study has both strengths and limitations. The strengths include the use of a nationally representative dataset, a clearly defined treatment framework, and robust econometric techniques to control for confounding, as well as conducting robustness checks and sensitivity analyses to provide credible evidence on the impact of ANC on adverse pregnancy outcomes. Limitations include the use of cross-sectional data limits our ability to establish causal relationships between ANC utilization and adverse pregnancy outcomes. Second, the reliance on self-reported data may introduce recall or reporting bias, particularly for sensitive health events. Third, while we employed matching techniques to minimize selection bias, unmeasured confounding variables—such as maternal nutrition, health-seeking behavior, or provider competency—may still influence the results. Future studies could consider using other approaches, such as the instrumental variable approach, endogenous switching regression, and longitudinal design, for further validation of our findings.

## Data Availability

The datasets presented in this study can be found in online repositories. The names of the repository/repositories and accession number(s) can be found below: https://dhsprogram.com/methodology/survey/survey-display-578.cfm.
