# Supplementary material for: Estimating the impact of prenatal health care services on adverse pregnancy outcomes in Tanzania: a propensity score matching approach
Source: Front Glob Womens Health. 2025 Jul 22;6:1589721. doi: 10.3389/fgwh.2025.1589721 (PMC12321850; doi:10.3389/fgwh.2025.1589721)
Supplement: Supplementary file 1 [file Table1.docx]

Supplementary Material

# Supplementary Figures and Tables.

## Supplementary Figures

Figure A.1 Kernel density distribution showing overlap and balancing between women with adequate ANC and without adequate ANC

 **Figure A.2: Kernel density distribution showing overlap and balancing between women with adequate ANC visits and with inadequate ANC visits**

Figure A.3: Kernel density distribution showing overlap and balancing between women with adequate ANC package and with inadequate ANC package

## Supplementary Tables

Table A.1 Summary statistics: ANC package

| **Variables** | **Pooled** | **ANC package=1** | **ANC package=0** | **p-value** |
| --- | --- | --- | --- | --- |
| ANC package (1=yes) | 0.322 (0.467) |  |  |  |
| Adverse Pregnancy Outcomes (1=yes) | 0.059 (0.236) | 0.015 (0.123) | 0.080 (0.271) | <0.01 |
| Woman's age (years) | 30.565 (6.475) | 30.185 (6.153) | 30.746 (6.616) | 0.013 |
| Women age square | 976.17 (413.14) | 948.98 (387.326) | 989.08 (424.) | 0.013 |
| Women's education (1=no educ) | 0.217 (0.412) | 0.141 (0.348) | 0.254 (0.435) | <0.01 |
| Women's education (2=primary educ) | 0.531 (0.499) | 0.516 (0.500) | 0.538 (0.499) | 0.163 |
| Women's education (3=sec or higher) | 0.252 (0.434) | 0.343 (0.475) | 0.209 (0.406) | <0.01 |
| Partner education (1=no educ) | 0.146 (0.353) | 0.096 (0.295) | 0.169 (0.375) | <0.01 |
| Partner education (2=primary educ) | 0.588 (0.492) | 0.529 (0.499) | 0.616 (0.486) | <0.01 |
| Partner education (3=sec or higher) | 0.266 (0.442) | 0.375 (0.484) | 0.215 (0.411) | <0.01 |
| Wealth status (1=poorest) | 0.222 (0.415) | 0.145 (0.352) | 0.258 (0.438) | <0.01 |
| Wealth status (2=poorer) | 0.202 (0.402) | 0.148 (0.356) | 0.228 (0.419) | <0.01 |
| Wealth status (3=Middle) | 0.199 (0.400) | 0.169 (0.375) | 0.214 (0.410) | <0.01 |
| Wealth status (4=Richer) | 0.195 (0.396) | 0.247 (0.431) | 0.171 (0.376) | <0.01 |
| Wealth status (5=Richest) | 0.182 (0.386) | 0.290 (0.454) | 0.130 (0.336) | <0.01 |
| Birth order | 0.554 (0.497) | 0.461 (0.499) | 0.599 (0.490) | <0.01 |
| Short birth interval | 0.223 (0.416) | 0.200 (0.400) | 0.234 (0.423) | <0.01 |
| Women employment (1=yes) | 0.661 (0.473) | 0.694 (0.461) | 0.646 (0.478) | <0.01 |
| Household head (1=male) | 0.869 (0.337) | 0.858 (0.349) | 0.874 (0.331) | 0.123 |
| Wife beating justified (1=yes) | 0.479 (0.500) | 0.442 (0.497) | 0.497 (0.500) | <0.01 |
| Decision making (1=yes) | 0.523 (0.500) | 0.554 (0.497) | 0.509 (0.500) | <0.01 |
| Media exposure (1=yes) | 0.640 (0.480) | 0.736 (0.441) | 0.594 (0.491) | <0.01 |
| Residence (1=rural) | 0.752 (0.432) | 0.606 (0.489) | 0.821 (0.383) | <0.01 |
| Community poverty (1=high) | 0.500 (0.500) | 0.348 (0.476) | 0.573 (0.495) | <0.01 |
| Community media access (1=high) | 0.466 (0.499) | 0.599 (0.490) | 0.402 (0.490) | <0.01 |
| Community literacy (1=high) | 0.380 (0.485) | 0.529 (0.499) | 0.309 (0.462) | <0.01 |
| Observations | 4665 | 1502 | 3163 |  |

Table A.2: Summary statistics: ANC Visits

| **Variables** | **Pooled** | **ANC Visits = 1** | **ANC Visits=0** | **p-value** |
| --- | --- | --- | --- | --- |
| ANC Visits (1=yes) | 0.322 (0.467) |  |  |  |
| Adverse Pregnancy Outcome (1=yes) | 0.059 (0.236) | 0.012 (0.107) | 0.012 (0.111) | 0.826 |
| Women age | 30.565 (6.475) | 30.294 (6.072) | 30.784 (6.696) | 0.013 |
| Women age square | 976.17 (413.14) | 954.58(386.14) | 992.48 (427.60) | 0.013 |
| Women's education (1=no educ) | 0.217 (0.412) | 0.149 (0.357) | 0.250 (0.433) | <0.01 |
| Women's education (2=primary educ) | 0.531 (0.499) | 0.545 (0.498) | 0.532 (0.499) | 0.462 |
| Women's education (3=sec or higher) | 0.252 (0.434) | 0.306 (0.461) | 0.219 (0.413) | <0.01 |
| Partner education (1=no educ) | 0.146 (0.353) | 0.112 (0.315) | 0.159 (0.365) | <0.01 |
| Partner education (2=primary educ) | 0.588 (0.492) | 0.564 (0.496) | 0.604 (0.489) | 0.018 |
| Partner education (3=sec or higher) | 0.266 (0.442) | 0.325 (0.468) | 0.237 (0.425) | <0.01 |
| Wealth status (1=poorest) | 0.222 (0.415) | 0.153 (0.360) | 0.251 (0.434) | <0.01 |
| Wealth status (2=poorer) | 0.202 (0.402) | 0.187 (0.390) | 0.208 (0.406) | 0.14 |
| Wealth status (3=Middle) | 0.199 (0.400) | 0.199 (0.400) | 0.197 (0.398) | 0.873 |
| Wealth status (4=Richer) | 0.195 (0.396) | 0.215 (0.411) | 0.190 (0.392) | 0.076 |
| Wealth status (5=Richest) | 0.182 (0.386) | 0.246 (0.431) | 0.154 (0.361) | <0.01 |
| Birth order | 0.554 (0.497) | 0.472 (0.499) | 0.592 (0.492) | <0.01 |
| Short birth interval | 0.223 (0.416) | 0.184 (0.388) | 0.208 (0.406) | <0.01 |
| Women employment (1=yes) | 0.661 (0.473) | 0.719 (0.450) | 0.642 (0.479) | <0.01 |
| Household head (1=male) | 0.869 (0.337) | 0.865 (0.342) | 0.869 (0.337) | 0.727 |
| Wife beating justified (1=yes) | 0.479 (0.500) | 0.445 (0.497) | 0.491 (0.500) | <0.01 |
| Decision making (1=yes) | 0.523 (0.500) | 0.562 (0.496) | 0.510 (0.500) | <0.01 |
| Media exposure (1=yes) | 0.640 (0.480) | 0.724 (0.447) | 0.602 (0.490) | <0.01 |
| Residence (1=rural) | 0.752 (0.432) | 0.697 (0.460) | 0.770 (0.421) | <0.01 |
| Community poverty(1=high) | 0.500 (0.500) | 0.419 (0.494) | 0.539 (0.499) | <0.01 |
| Community media access (1=high) | 0.466 (0.499) | 0.513 (0.500) | 0.444 (0.497) | <0.01 |
| Community literacy (1=high) | 0.380 (0.485) | 0.444 (0.497) | 0.347 (0.476) | <0.01 |
| Observations | 4665 | 1,118 | 2,804 |  |

Table A.3: Common support

| **Assigned treatment** | **Off support** | **On support** | **Total** |
| --- | --- | --- | --- |
| Inadequate ANC package  Full ANC package  Total | 0  1  1 | 3,163  1,501  4,664 | 3,163  1,502  4,665 |
| Inadequate ANC visits  Adequate ANC visits  Total | 0  2  2 | 2,804  1,116  3,920 | 2,804  1,118  3,922 |
| Adequate ANC  Inadequate ANC  Total | 0  0  0 | 4,080  578  4,658 | 4,080  578  4,658 |
